# Public availability of results of observational studies evaluating an intervention registered at ClinicalTrials.gov

**DOI:** 10.1186/s12916-016-0551-4

**Published:** 2016-01-28

**Authors:** Marie Baudart, Philippe Ravaud, Gabriel Baron, Agnes Dechartres, Romana Haneef, Isabelle Boutron

**Affiliations:** Centre d’Épidémiologie Clinique, Hôpital Hôtel Dieu, Assistance Publique des Hôpitaux de Paris, 1, Place du parvis Notre Dame, 75181 Paris, Cedex 4 France; Paris Descartes University, Paris, France; METHODS Team, Centre of Research in Epidemiology and Statistics Sorbonne Paris Cité, UMR 1153, INSERM, Paris, France; French Cochrane Center, Paris, France; Department of Epidemiology, Columbia University Mailman School of Public Health, New York, NY USA

**Keywords:** Observational studies, Trial registration, Waste in research

## Abstract

**Background:**

Observational studies are essential for assessing safety. The aims of this study were to evaluate whether results of observational studies evaluating an intervention with safety outcome(s) registered at ClinicalTrials.gov were published and, if not, whether they were available through posting on ClinicalTrials.gov or the sponsor website.

**Methods:**

We identified a cohort of observational studies with safety outcome(s) registered on ClinicalTrials.gov after October 1, 2007, and completed between October 1, 2007, and December 31, 2011. We systematically searched PubMed for a publication, as well as ClinicalTrials.gov and the sponsor website for results. The main outcomes were the time to the first publication in journals and to the first public availability of the study results (i.e. published or posted on ClinicalTrials.gov or the sponsor website). For all studies with results publicly available, we evaluated the completeness of reporting (i.e. reported with the number of events per arm) of safety outcomes.

**Results:**

We identified 489 studies; 334 (68 %) were partially or completely funded by industry. Results for only 189 (39 %, i.e. 65 % of the total target number of participants) were published at least 30 months after the study completion. When searching other data sources, we obtained the results for 53 % (n = 158; i.e. 93 % of the total target number of participants) of unpublished studies; 31 % (n = 94) were posted on ClinicalTrials.gov and 21 % (n = 64) on the sponsor website. As compared with non-industry-funded studies, industry-funded study results were less likely to be published but not less likely to be publicly available. Of the 242 studies with a primary outcome recorded as a safety issue, all these outcomes were adequately reported in 86 % (114/133) when available in a publication, 91 % (62/68) when available on ClinicalTrials.gov, and 80 % (33/41) when available on the sponsor website.

**Conclusions:**

Only 39 % of observational studies evaluating an intervention with safety outcome(s) registered at ClinicalTrials.gov had their results published at least 30 months after study completion. The registration of these observational studies allowed searching other sources (results posted at ClinicalTrials.gov and sponsor website) and obtaining results for half of unpublished studies and 93 % of the total target number of participants.

**Electronic supplementary material:**

The online version of this article (doi:10.1186/s12916-016-0551-4) contains supplementary material, which is available to authorized users.

## Background

Failure to provide access to research results is a key source of wasted research [1]. The results for more than 50 % of clinical trials are never published, and publication is more likely for clinical trials with statistically significant (positive) than negative results [2–6]. Lack of the availability of research findings has serious consequences; it affects the results of systematic reviews and meta-analyses and distorts the evidence used for the prioritization of research questions and clinical and policy decision-making [7–9]. In response to this waste, in 2005, the International Committee of Medical Editors required the registration of all clinical trials, before study inception, in a publicly accessible register such as ClinicalTrials.gov [10, 11]. In 2007, the US Food and Drug Administration Amendments Act also required the posting of clinical trials results for all phase II to IV trials of drugs, biologic treatments and devices having at least one site in the United State at ClinicalTrials.gov no later than 1 year after the date of final collection of data for the pre-specified primary outcome [12]. In Europe, a new law that will be implemented in 2016 will require that all clinical trials be registered on a publicly accessible European Union clinical-trials register before they can begin, with a summary of trial results posted within a year after the end of the trial.

These policies have an important impact and allowed increase research value. In fact, thanks to trials registration, unpublished studies are identified, and their results could be made available through posting. However, registration is currently mandatory only for clinical trials.

Observational studies such as cohort and case–control studies are important for assessing the intervention effect [13–16]. They are particularly useful designs when randomized controlled trials (RCTs) are not feasible or when assessing rare adverse events and long-term effectiveness. Such studies represent a large part of the published literature and outnumber published RCTs [17]. Nevertheless, prospective registration of observational studies is not currently requested [18]. Despite not being mandatory, more than 35,000 observational studies are registered at ClinicalTrials.gov.

Our hypothesis is that registration of observational studies evaluating an intervention in ClinicalTrials.gov is important to increase research value as it allows identifying unpublished studies and obtaining unpublished results.

The main objectives of this study were (1) to evaluate whether results of observational studies evaluating an intervention with safety outcome(s) registered at ClinicalTrials.gov were published and, if not, whether they were available through posting on ClinicalTrials.gov or the sponsor website; (2) to evaluate and compare the time to publication and to public availability of results after searching other sources by study funding source; and (3) to evaluate the completeness of reporting of the outcomes designated as safety issues.

## Methods

We identified a cohort of observational studies evaluating an intervention with safety outcome(s) registered at ClinicalTrials.gov.

### Search for relevant studies

We searched ClinicalTrials.gov on April 14, 2014, by using “completed” for recruitment, “observational studies” for study type, and date of first registration between October 1, 2007, and December 31, 2011, and “has an outcome measure designated as a safety issue” in the safety issue field of ClinicalTrials.gov. We chose October 2007 because modifications were made at this time to the design-specific data elements used for registering observational studies on ClinicalTrials.gov. These changes were strongly influenced by protocol-related items in the Strengthening the Reporting of Observational Studies in Epidemiology (STROBE) statement [13].

Observational studies are defined by ClinicalTrials.gov as studies where “*investigators assess health outcomes in groups of participants according to a protocol or research plan. Participants may receive interventions, which can include medical products, such as drugs or devices, or procedures as part of their routine medical care, but participants are not assigned to specific interventions by the investigator (as in a clinical trial *https://clinicaltrials.gov/ct2/about-studies/glossary#O).”

### Identification of relevant observational studies

Among the studies retrieved, we identified all observational prospective studies assessing interventions that had a primary completion date (i.e. the date when the final patient was examined or received an intervention for the purposes of final collection of data for the primary outcome) between October 1, 2007, and December 31, 2011. We defined interventions as all pharmacologic and non-pharmacologic treatments (pharmaceutical drugs, surgery, education, rehabilitation, etc.) aimed at improving the participants’ health. We chose this time period to be able to investigate public availability (i.e. at least 24 months after the study primary completion). We excluded studies with a primary completion date after December 2011, studies assessing genetics, predictors, or risk factors; not assessing interventions, pharmacodynamics, and pharmacokinetics of drugs; phase 0, I, II, I/II and II/III studies; studies with healthy volunteers, and retrospective and randomized studies. We also excluded studies for which the primary completion date was not reported at ClinicalTrials.gov. The selection process was performed by one researcher, and all records included were independently verified by a second researcher.

### Extraction of data from ClinicalTrials.gov

We downloaded from ClinicalTrials.gov the following data concerning the characteristics of the studies: clinical trial number (NCT), title, study design (defined with the observational model: cohort, case–control, case-only, case crossover or other; and time perspective: prospective, cross-sectional or other), enrollment (i.e. sample size), first received date, primary completion date, results first received date, condition and intervention under study, outcome measures, locations of recruitment and funding source. The funding source was classified at ClinicalTrials.gov as “NIH” (National Institutes of Health), “US federal”, “industry”, and other non-industry organizations (universities, hospitals, foundations, and other government and other non-industry organizations). We secondarily categorized funding sources as non-industry (i.e. funded by NIH, US federal, other non-industry organizations) or industry (i.e. partially or totally funded by industry).

One researcher classified the following information from the full ClinicalTrials.gov record: medical field, type of intervention (drug, device, procedure/surgery, other), location of recruitment (Europe, North America, South America, Africa, Asia, Oceania) and study purpose as safety, efficacy or both. As a quality assurance procedure, a second researcher independently verified 50 % of the data.

### Publication of study results in journals

For each observational study identified, we systematically searched for a publication reporting the study results (search date June 2014). First, we examined the “publication” field at ClinicalTrials.gov to search for a citation for an article that described the study results. If no citation was reported, we searched MEDLINE via PubMed by using the ClinicalTrials.gov identification number (NCT). If no publication was identified, we searched MEDLINE via PubMed by using keywords for the intervention under study and the condition. A researcher screened all citations retrieved up to the primary completion date registered at ClinicalTrials.gov and selected all citations corresponding to the selected study. A second researcher independently performed the search on PubMed for all the studies for which no publication was identified; any discrepancies were discussed until obtaining consensus.

Finally, if no publication was identified, we contacted the sponsor or the principal investigator. We searched in the “additional information” field of ClinicalTrials.gov for a link to the sponsor website for contacting the sponsor. If no link was available, we recorded the principal investigator’s name from the “contacts and locations” fields of ClinicalTrials.gov and searched PubMed and Google to identify their email address. The email reminded the recipient of the study NCT number and inquired about the study publication, presentation at a congress, and plans for future publication (Additional file 1: Appendix 1). We systematically sent two reminders. If no answer was received, the study findings were considered unpublished. When the study results were reported as an abstract or poster presented at a scientific meeting, we considered that the results were not available. In fact, previous evidence showed that the quality of abstracts presented at meetings is suboptimal, frequently including results that are not the final results [19, 20].

To determine whether the publication(s) corresponded to the registered observational study, we retrieved the full-text article for all citations selected and assessed from the abstract and the full text, if needed, whether a combination of information, including description of interventions and conditions, population, location, responsible party, number of participants, primary outcome measures, and primary funding sponsor partly or completely matched the information at ClinicalTrials.gov. We only selected articles that reported the results of the study. All cases were assessed by a second independent researcher, and disagreements were resolved by consensus*.*

If the publication highlighted that the study did not actually fulfill the inclusion criteria, while it was impossible to detect it from ClinicalTrials.gov record, the studies were still included in the analysis and considered as published to avoid bias. This occurred for eight studies (randomized n = 6; retrospective n = 1; phase 0/I/II n = 1).

### Search of results posted on ClinicalTrials.gov and the sponsor website

For each observational study identified, we assessed whether unpublished results were publicly available from other sources. For this purpose, (1) we searched whether results were posted at ClinicalTrials.gov and (2) systematically searched the sponsor website to identify whether the results were available. For this, we searched ClinicalTrials.gov for a link to the sponsor website. If no link was available, we searched Google using the sponsor name to identify the sponsor website. Then, we searched the website for a section dedicated to access to study results and used the study NCT number to find the study results.

### Main outcomes

The main outcomes were the time to the first publication in journals, and the time to the first public availability of the study results.

The time to the first publication in journals was the time (in months) that elapsed between the primary completion date of the study and the publication. The study primary completion date was obtained from ClinicalTrials.gov. The publication date was the first date an article was made available online ahead of print (i.e. epub date indexed on PubMed) or published in a paper-printed version.

The time to the first public availability of the study results was the time (in months) between the primary completion of the study and the first public availability of the study results by publication or posting on ClinicalTrials.gov or the sponsor website. When results were available in different sources, we used the first date.

Secondary outcomes were the proportion of study results publicly available (via publication or posting on ClinicalTrials.gov or the sponsor website) at 12 and 24 months after primary completion.

### Completeness of reporting of outcomes designated as safety issues

For all studies with results publicly available (via publication or posting on ClinicalTrials.gov or the sponsor website), we evaluated the completeness of reporting of all primary outcome measure(s) designated as a “safety issue” and when not available, all secondary outcome measure(s) designated as a safety issue as recorded from ClinicalTrials.gov. Then, for each outcome recorded, we systematically searched in the report available (i.e. publication, and for unpublished studies, results posted on ClinicalTrials.gov or the sponsor website) whether the results were adequately reported (i.e. reported with the number of events per arm), partially reported (i.e. reported with the number of events pooled or only mentioned), or not reported. When several publications were available, we selected the publication with the results more completely reported.

For all studies with results publicly available, we determined the proportion of studies with all primary outcomes designated as safety issues adequately reported and when not available, the proportion of studies with all secondary outcomes adequately reported.

For this analysis, we excluded studies when the results were not published or available in English.

### Statistical analysis

Quantitative variables are described with median (quartile 1–3; Q1–Q3) and qualitative variables with number and percentages. For each outcome, we assessed Kaplan-Meier estimates of the cumulative incidence of studies (with 95 % confidence interval (CI)) at 12 and 24 months. All studies without results available in the different sources were censored on June 1, 2014 (i.e. search date). Cumulative incidence curves estimated by Kaplan–Meier methods are displayed globally and by funding source (partially or completely industry-funded; non-industry-funded). Univariate and multivariate Cox proportional hazards regression analyses were used to calculate adjusted hazard ratios (HRs) by funding source (with 95 % CIs and *P* values from Wald test)*.* The following confounding variables were entered in the multivariate Cox model: type of intervention (drug, device, procedure/surgery, or other), location recruitment (Africa/Asia/South America or Europe/Australia/North America), objective of the study (safety or both efficacy and safety, or only efficacy), sample size and registration period (before the start date of the study/between the start date of the study and the primary study completion/after the primary study completion date).

Statistical analysis involved use of SAS v9.4 (SAS Inst. Inc., Cary, NC) and R software (v3.1.2) (http://www.R-project.org, the R Foundation for Statistical Computing, Vienna, Austria).

## Results

### Study selection and characteristics

**Fig. 1:**
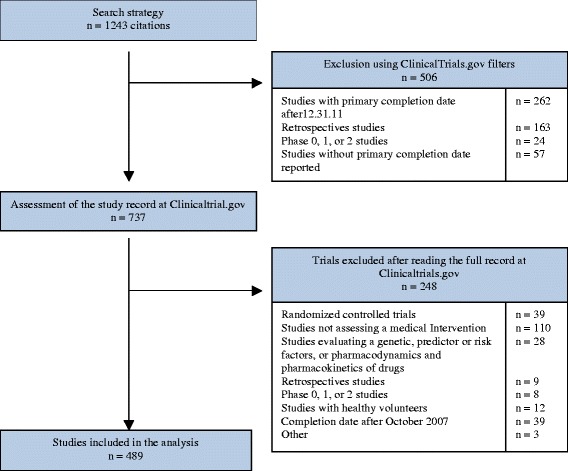
Selection of observational studies

From the 1,243 citations screened, 489 studies were selected (Fig. 1). The characteristics of the selected studies are shown in Table 1. Only about one-quarter of the studies were registered before the start date of the study; 30 % were registered after the study primary completion date. Overall, 333 studies (68 %) were conducted in high-income countries, 111 (23 %) with at least one site in North America, and 68 % (n = 334) were partially or completely funded by industry. The median (Q1–Q3) sample size was 300.0 (75.0–1045.0); 507.0 (131.0–1794.0) for studies with industry funding and 80.0 (32–219.0) for those with non-industry funding. The cumulative target enrollment for all studies was 1,214,218 participants. The study objective in 72 % (n = 351) of studies was to assess safety.

**Table 1:** Characteristics of selected studies (n = 489) by funding: industry or non-industry

Characteristics		Industry funded (totally or partially) (n = 334)	Non-industry funded (n = 155)	Total (n = 489)
Registration	Before the start date of the study	69 (20.7)	43 (27.7)	112 (22.9)
	Between the start date of the study and study primary completion date	174 (52.1)	58 (37.4)	232 (47.4)
	After the study primary completion date	91 (27.2)	54 (34.8)	145 (29.7)
Medical field	Cardiovascular	51 (15.3)	27 (17.4)	78 (16.0)
	Endocrinology	61 (18.3)	4 (2.6)	65 (13.3)
	Infectious diseases	46 (13.8)	16 (10.3)	62 (12.7)
	Urology/gynecology	30 (9.0)	13 (8.4)	43 (8.8)
	Rheumatology	23 (6.9)	2 (1.3)	25 (5.1)
	Bronchopulmonary	20 (6.0)	9 (5.8)	29 (5.9)
	Neurology	18 (5.4)	6 (3.9)	24 (4.9)
	Oncology	12 (3.6)	10 (6.5)	22 (4.9)
	Gastroenterology	11 (3.3)	19 (12.3)	30 (6.1)
	Ophthalmology	10 (3.0)	9 (5.8)	19 (3.9)
	Psychiatry	10 (3.0)	2 (1.3)	12 (2.5)
	Other	42 (12.6)	38 (24.5)	80 (16.4)
Location	Africa/Asia/South America	114 (34.1)	42 (27.1)	156 (31.9)
	Europe/Australia/North America	220 (65.9)	113 (72.9)	333 (68.1)
Intervention	Drug	278 (83.2)	52 (33.5)	330 (67.5)
	Device	43 (12.9)	45 (29.0)	88 (18.0)
	Procedure/surgery	9 (2.7)	54 (34.8)	63 (12.9)
	Other	4 (1.2)	4 (2.6)	8 (1.6)
Sample size	≤100	68 (20.4)	94 (60.6)	162 (33.1)
	100–500	95 (28.4)	40 (25.8)	135 (27.6)
	>500	171 (51.2)	21 (13.5)	192 (39.3)
Study objective	Safety or both safety and efficacy	279 (83.5)	72 (46.5)	351 (71.8)
	Efficacy	55 (16.5)	83 (53.5)	138 (28.2)

Data are presented as number (%)

### Publication of study results

The median time between the study primary completion date and our search was 49.0 (Q1–Q3, 41.0–62.0) months; for all studies, at least 30 months had elapsed since the study primary completion date. Among the 489 studies, we identified a publication via the citation reported at ClinicalTrials.gov for 75 and via a systematic search of PubMed for 99. For the 314 remaining studies without a publication identified, we obtained an email address and contacted the sponsor/principal investigator of 241 studies, of which 52 responded and 15 provided an article (Additional file 1: Appendix 2).

**Fig. 2:**
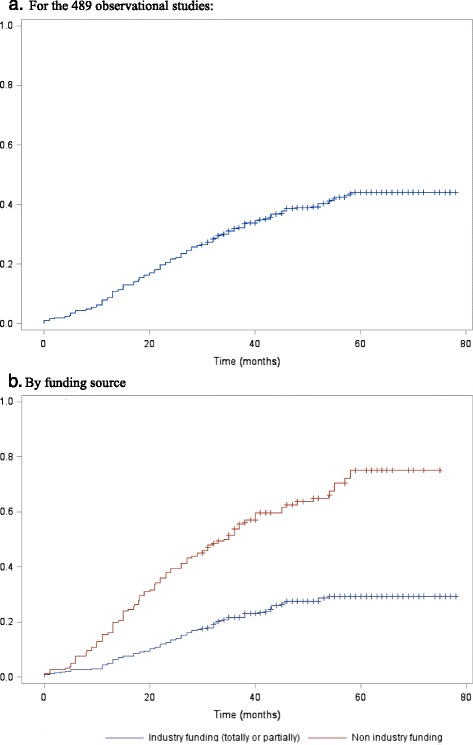
Probability of first publication in journals by funding source for published articles indexed in PubMed  for **a**) the whole sample of 489 observational studies and **b**) stratified by funding source

Overall, results of only 189 (39 %) studies were published at least 30 months after study primary completion, corresponding to a cumulative target of enrollment of 785,437 participants (65 % of the total target number of participants). The global cumulative percentage of studies with results published over time and stratified by funding source are in Fig. 2. The cumulative percentage of observational studies with results published 12 months after primary completion was 8.2 % (95 % CI, 5.7–10.6): 4.8 % (95 % CI, 2.5–7.1) of industry-funded studies and 15.5 % (9.8–21.2) of non-industry-funded studies. At 24 months, 21.3 % (17.6–24.9) of studies were published: 13.2 % (9.5–16.8) of industry-funded studies and 38.7 % (31.0–46.4) of non-industry-funded studies. After adjusting for confounding variables (i.e. type of intervention, location recruitment, objective of the study, sample size and registration period), industry-funded study results were less likely to be published than non-industry funded studies (adjusted HR, 0.39; 95 % CI, 0.27–0.56; *P* <0.0001).

### Posting of study results on ClinicalTrials.gov and on the sponsor website

When searching the results section of ClinicalTrials.gov, we obtained the results for 31 % (n = 94) of our unpublished studies (i.e. 147,593 cumulative target participants). When searching sponsor websites, we obtained the results for 64 (21 %) of unpublished studies (i.e. 195,291 cumulative target participants), of which 39 could be obtained through a link to the sponsor website available in ClinicalTrials.gov. All study results obtained from sponsor websites were sponsored by Bayer (n = 16), GSK (n = 12), or Novo Nordisk (n = 36).

### Overall public availability of study results

**Fig. 3:**
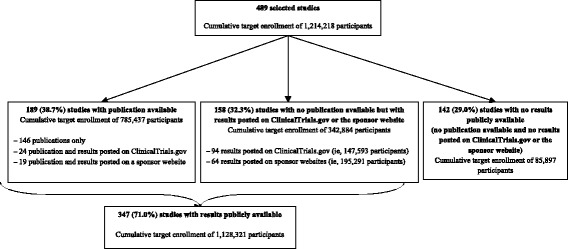
Availability of study reports at least 30 months after study completion

Figure 3 summarizes the public availability of results of the selected studies in terms of publication or posting on ClinicalTrials.gov or the sponsor website. Overall, 347 studies (71 %) had results publicly available at least 30 months after study primary completion, corresponding to a cumulative target of enrollment of 1,128,321 participants (i.e. 93 % of the total target number of participants). The median (Q1–Q3) sample size of studies with publication available was 165 (60–882), studies with unpublished data available through ClinicalTrials.gov, 503 (154–1307) studies with unpublished data available through the sponsor website 1001 (393–2366), and studies with no results publicly available 130 (50–556).

**Fig. 4:**
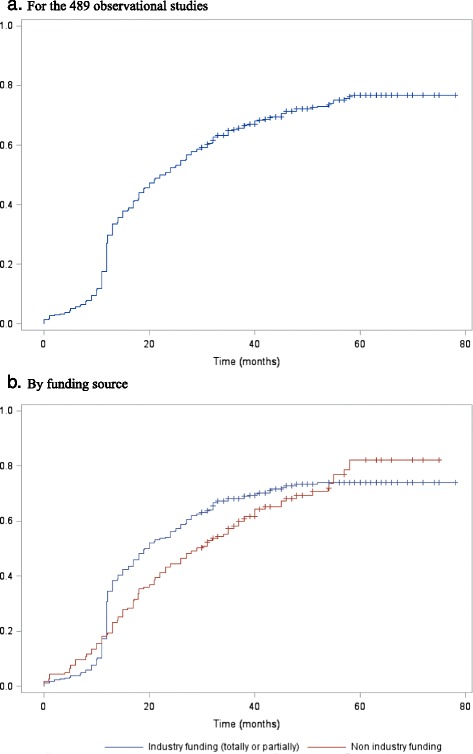
Overall public availability of study results (publication available or results posted on ClinicalTrials.gov or the sponsor website) and by funding source

The cumulative proportion of studies with results publicly available over time overall and stratified by funding source is shown in Fig. 4. Overall, the cumulative percentage of studies with results publicly available was 27.0 (23.1–30.9) at 12 months and 51.7 (47.3–56.2) at 24 months; the proportion for industry- and non-industry-funded studies was 30.8 % (25.9–35.8) versus 18.7 % (12.6–24.8) at 12 months and 55.4 % (50.1–60.7) versus 43.9 % (36.0–51.7) at 24 months. After adjusting for confounding variables, the probability of having results publicly available did not differ between industry-funded and non-industry-funded studies (adjusted HR, 1.16; 95 % CI, 0.88–1.52; *P* = 0.30). Additional file 1: Appendix 3 reports the availability of the study results according to the date of registration (before the start of the study, between the start and the study completion, after the study completion). Overall, 43 % of the studies that were registered after the study primary completion date were published, 33 % were not published but had their data available in other sources, and 23 % had no data available.

### Completeness of reporting (Table 2)

Of the 347 studies with results publicly available, 243 reported at least one primary outcome measure designated as a “safety issue”. We evaluated the completeness of reporting of 242 studies (one excluded because not published in English): all these outcomes were adequately reported in 86 % (114/133) when available in a publication, 91 % (62/68) when available on ClinicalTrials.gov, and 80 % (33/41) when available on the sponsor website. Of the 96 studies that did not have a primary outcome measure designated as a “safety issue”, 66 (69 %) adequately reported all secondary outcomes designated as a safety issue; 60 % when available in a publication, 92 % when available on ClinicalTrials.gov, and 61 % when available on the sponsor website.

**Table 2:** Completeness of reporting of primary outcomes and secondary outcomes designated as a “safety issue”

	Published articles^a^	Results not published but posted on ClinicalTrials.gov	Results not published but posted on the sponsor website	Total
Completeness of reporting of primary outcome(s) designated as a safety issue	n = 133	n = 68	n = 41	n = 242
All outcomes adequately reported	114 (85.7)	62 (91.2)	33 (80.5)	209 (86.4)
At least one outcome partially reported or not reported	9 (6.8)	2 (2.9)	6 (14.6)	17 (7.0)
All outcomes not reported	10 (7.5)	4 (5.9)	2 (4.9)	16 (6.6)
Completeness of reporting of secondary outcome(s) designated as a safety issue for trials with no primary outcomes designated as a safety issue	n = 47	n = 26	n = 23	n = 96
All outcomes adequately reported	28 (59.6)	24 (92.4)	14 (60.9)	66 (68.7)
At least one outcome partially reported or not reported	10 (21.3)	1 (3.8)	8 (34.8)	19 (19.8)
No outcome reported	9 (19.1)	1 (3.8)	1 (4.3)	11 (11.5)

Data are presented as number (%)

^a^One missing value

## Discussion

We evaluated the public availability of study results in a cohort of 481 observational studies evaluating an intervention with safety outcome(s) registered at ClinicalTrials.gov and completed for more than 30 months. Only 39 % (n = 189) had results published, corresponding to 65 % of the total target number of participants. The cumulative percentage of studies with results published at 12 and 24 months after primary completion were 8.2 % (95 % CI, 5.7–10.6) and 21.3 % (17.6–24.9), respectively. However, when searching other data sources (results posted on ClinicalTrials.gov and sponsor websites), we obtained the results for about half of the studies with unpublished results (n = 158, 53 %) corresponding to 93 % of the total target number of participants. Further, the median sample size of unpublished results posted on ClinicalTrials.gov and sponsor websites was high.

To our knowledge, this is the first large study evaluating the public availability of the results of observational studies registered at ClinicalTrials.gov in terms of publication or posting on ClinicalTrials.gov or the sponsor’s website. Most evidence of the lack of availability of research results has focused on the publication of clinical trials and the posting of results on ClinicalTrials.gov [5, 6, 21–24]. Large cohorts of registered clinical trials showed that the results of only 46–63 % are published [21, 23]. In the field of diagnostic studies, results for 54 % of studies completed for at least 18 months were published [25].

Our study has several important implications; they clearly illustrate the need for a change in policy with a request to also prospectively register observational studies. In many situations, observational studies are the only data available because RCTs are not appropriate or feasible. The number of published meta-analyses including observational studies in health has increased substantially and these meta-analyses could be used to inform clinical decision-making and public health policy [18, 26]. Registration of observational studies is debated, as shown by recent editorials published in major medical journals [17, 27–32]. However, much of the rationale for the prospective registration of clinical trials [18, 33] also applies to the registration of observational studies. Although registration at ClinicalTrials.gov does not guarantee that trial results will be published in a timely manner [22, 34–36], it allows knowing the existence of the study, searching for unpublished data, exploring publication bias, outcome reporting bias, and fidelity to the protocol [37, 38].

Our results also highlight the need to reconsider the strategy used to identify research findings. In fact, searching the results section at ClinicalTrials.gov as well as the sponsor website increased by two-fold the number of studies with results available and allowed access to the data of 93 % of the total target number of participants. It is consequently very important that systematic reviewers search for these data in trials registries and the sponsors websites. Previous studies comparing the results posted and published for clinical trials showed that results are more completely reported in registries than in publications [21, 23] and that discrepancies between the ClinicalTrials.gov results database and matching publications are common [38]. However, it is unclear which source is more accurate [38].

Publication of research findings in peer-reviewed journals is considered essential for disseminating research results. However, the publication process is long, complicated and supposes an important investment from the sponsor and investigator. Lack of or delayed publication could be related to the lack of incentives to disseminate negative results, time constraints, limited resources, changing interests, or difficulties and failure to have the results published [34]. The sponsor may prefer posting results on a website than investing in publication in a peer-reviewed journal. However, we question why sponsors post results on their website and not on ClinicalTrials.gov. In fact, ClinicalTrials.gov performs a quality control, which is not the case with the sponsor website.

Finally, ClinicalTrials.gov offers researchers the opportunity to provide access to their data if they decide not to publish them in a peer-reviewed journal. For clinical trials, the posting of results is a requirement. The World Health Organization is calling for a strict timeline for public disclosure of clinical trial results and published a new Statement on the Public Disclosure of Clinical Trial Results, which specifies that study results be reported at least 30 months after a study is completed. This requirement should also be extended to observational studies.

Our study has some potential limitations. First, as registration of observational studies is not mandatory, we could explore the public availability of the trial results only for observational studies registered at ClinicalTrials.gov. However, the publication rate and public availability of results is not likely to be higher for observational studies that were not registered. Second, our search was performed only in Medline via PubMed and we may have missed some publications. However, Medline is the largest database of biomedical journals and is the source that nearly all physicians and policymakers use to access clinical trial findings. Further, a recent study showed that searching Embase has a modest impact on the results of systematic reviews [39] and most studies evaluating RCT publication did not search Embase [21, 34]. Additionally, we systematically contacted the sponsor or investigator to check whether the study was published. Finally, we used the data recorded at ClinicalTrials.gov, but this information is not always accurate, and ClinicalTrials.gov added a database of summary results allowing for reporting results of observational studies according to the STROBE statement only in September 2008.

## Conclusion

In conclusion, about 39 % of observational studies evaluating an intervention with a safety outcome and registered at ClinicalTrials.gov had their results published. Searching for unpublished data allowed for access to more than two-thirds of the study results and 93 % of the total target number of participants. Given the potential important benefit of requesting the registration of observational studies, this practice should be required by research regulations.

### Availability of data and materials

The data will be made available on Dryad.

## Additional file

Additional file 1:
**Appendix 1:** Emails for principal investigators indexed in ClinicalTrials.gov. **Appendix 2:** Flowchart of email responses. **Appendix 3:** Publication status according to the date of registration (before the start of the study, between the start and the study completion, after the study completion). (DOCX 31 kb)
