# Supplementary material for: Public availability of results of observational studies evaluating an intervention registered at ClinicalTrials.gov
Source: BMC Med. 2016 Jan 28;14:7. doi: 10.1186/s12916-016-0551-4 (PMC4730754; doi:10.1186/s12916-016-0551-4)
Supplement: Additional file 1: — Appendix 1: Emails for principal investigators indexed in ClinicalTrials.gov. Appendix 2: Flowchart of email responses. Appendix 3: Publication status according to the date of registration (before the start of the study, between the start and the study completion, after the study completion). (DOCX 31 kb) [file 12916_2016_551_MOESM1_ESM.docx]

**APPENDICES**

**Appendix 1. Emails for principal investigators indexed inClinicalTrials.gov**

**Object :** Inquiry about the study XXXXXXXXX (NCT999999999999 on ClinicalTrials.gov)

| Dearcolleague, |
| --- |
| We are very interested by your study entitled XXXXXXXXX registered on ClinicalTrials.gov, NCT999999999999. |
| We would be very grateful if you could tell us whether this study has been published and whether we could obtain a reprint. If it has not been published yet could you tell us whether the results have been presented in a congress and how we could access the results and what are your plans for future publication. |
| Thank you for your help. |
| Best regards, |
| Marie Gauthier, Pr Isabelle BOUTRON  Centre d’Epidémiologie Clinique Université Paris Descartes INSERM U1153 French Cochrane center Hôpital Hôtel Dieu, Aile A2 1er étage 1, Place du parvis Notre Dame 75181 Paris Cedex 4 E-mail : [isabelle.boutron@htd.aphp.fr](mailto:isabelle.boutron@htd.aphp.fr) Tel : 01 42 34 78 33 Fax : 01 42 34 87 90 |

**Appendix 2: Flowchart of email responses**

n = 318

Emails sent to sponsor/principal investigators

n = 241

Observational studies without articles found on PubMed

n = 37

n = 15

Article provided

Answer that the study was not published

No answer by June 30, 2014

n = 82

n = 73

Automatic answer

n = 34

False email address

n = 77

No email addressidentified

**Appendix 3 : Publication status according to the date of registration (before the start of the study, between the start and the study completion, after the study completion)**

We report below the number of studies registered before the start date of the study, between the start date of the study and study primary completion date, and after the study primary completion date according to the publication status and access to data in other sources. Overall, 43% of the studies that were registered after the study primary completion date were published, 33% were not published but had their data available in other sources, and 23% had no data available. We cannot make any assumption regarding the reason for retrospective registration.

|  | **Studies with publication available** | **Studies with unpublished data available** | **Studies with no results publicly available** |
| --- | --- | --- | --- |
| **Before the start date of the study (n=112)** | 44 (39.3) | 30 (26.8) | 38 (33.9) |
| **Between the start date of the study and the study completion (n=232)** | 82 (35.3) | 80 (34.5) | 70 (30.2) |
| **After the study completion (n=145)** | 63 (43.4) | 48 (33.1) | 34 (23.5) |
